# Stakeholder perceptions about the establishment of medical simulation-based learning at a university in a low resource setting: a qualitative study in Uganda

**DOI:** 10.1186/s12909-020-02301-3

**Published:** 2020-10-22

**Authors:** Josephine Nambi Najjuma, Francis Bajunirwe, Margaret Twine, Tamara Namata, Catherine Kalimba Kyakwera, Moses Cherop, Data Santorino

**Affiliations:** 1 Department of Nursing, Mbarara University of Science and Technology, P.O.BOX 1410 Mbarara, Uganda; 2 Simulation Center, Mbarara University of Science and Technology, P.O.BOX 1410 Mbarara, Uganda; 3 Department of Community Health, Mbarara University of Science and Technology, P.O Box 1410, Mbarara, Uganda; 4 Department of Pediatrics and Child Health, Mbarara University of Science and Technology, P. O Box 1410, Mbarara, Uganda

**Keywords:** Medical simulation, Low and middle-income country, Simulation center, Stakeholders

## Abstract

**Background:**

Simulation based learning (SBL) is a technique where teachers recreate “real life” clinical experiences for health care teams for purposes of gaining clinical skills in a safe environment. There is evidence that SBL is superior to the traditional clinical teaching methods for acquisition of clinical skills. Although it is well established in resource rich settings, there is limited experience in resource limited settings and there is uncertainty regarding how SBL will be perceived among the stakeholders in medical education. As part of the steps leading to implementation of a SBL program at a university in Uganda, we sought to describe the perceptions of various stakeholders regarding the introduction of SBL methodology into learning at a medical school in Uganda.

**Methods:**

We conducted a formative qualitative assessment using key informant interviews (KIIs) among faculty members and university administrators and focus group discussions (FGDs) among medical and nursing students at Mbarara University of Science and Technology. Data were collected till saturation point and were transcribed and analyzed manually using open and axial coding approaches to develop themes.

**Results:**

We conducted seven KIIs and three FGDs. Overall, findings were categorized into five broad themes: 1. Motivation to adopt simulation-based learning 2. Prior experience and understanding of simulation-based education 3. Outcomes arising from introduction of medical simulation 4. Drawbacks to establishment of medical simulation; and 5. Potential remedies to the drawbacks. Overall, our data show there was significant buy-in from the institution for SBL, stakeholders were optimistic about the prospects of having a new method of teaching, which they perceived as modern to complement the traditional methods. There was significant knowledge but very limited prior experience of medical simulation. Also, there was some concern regarding how students and faculty would embrace training on lifeless objects, the human resources needed and sustainability of simulation-based learning in the absence of external funding.

**Conclusion:**

Stakeholders perceive SBL positively and are likely to embrace the learning methods. Concerns about human resource needs and sustainability need to be addressed to ensure acceptability.

## Background

Simulation based learning (SBL) is a technique where teachers recreate “real life” clinical experiences for health care teams for purposes of gaining clinical skills in a safe environment [1, 2]. SBL provides the opportunity for immediate feedback thus enhancing and promoting experiential learning. There is evidence that SBL is superior to the traditional clinical teaching methods for acquisition of a wide range of skills [3]. There is also growing evidence to show that simulation-based learning is superior to problem-based learning for the acquisition of critical assessment and management skills, particularly for clinical emergencies [4, 5]. During SBL scenarios, manikins are used to simulate real medical conditions for learners to work in teams to manage the condition like they would in “real life”. Thereafter, the facilitator guides the post scenario discussion for learners to share frames of practice and hence learning [1].

Although the effectiveness of SBL has been demonstrated [6], almost all the experiences are from resource rich settings. This teaching methodology is new in sub Saharan Africa and there is a need to examine its potential in these new settings. There is keen interest to introduce SBL in sub Saharan Africa, but there is concern that its introduction may be met with skepticism regarding its effectiveness or the capability of institutions to implement it.

The population in sub Saharan Africa is rapidly growing and so is the demand for more health workers. To meet these demands, medical schools are increasing their intake of medical students. However, the training infrastructure and staffing are not growing at the same rate. The first step to provide better quality of care begins with how healthcare professionals are trained [7]. Due to the growing number of medical students, there is limited opportunity for them to apply theory into clinical practice, and this may lead to the overall feeling of unpreparedness to handle patients [8]. Globally, health care education is evolving towards a competence-based approach [9]. These needs have been addressed by adopting and implementing innovative teaching technologies like SBL [7].

The Faculty of Medicine at Mbarara University and partners from Stavanger Acute Medicine Foundation for Education and Research (SAFER), Norway and University of Calgary, Canada collaborated to establish a simulation center of excellence at Mbarara University of Science and Technology (MUST). This Simulation Center at MUST is the first of the kind in the East African region. The main aim of the partnership was to establish a regional center of excellence in simulation training, research and skills enhancement among pre service and in-service health personnel mainly in maternal and child health and also support other clinical departments to adopt for simulation teaching.

Prior to the implementation of SBL, and in the early phases of implementation, we sought to understand the perception of key stakeholder towards SBL. Given the novelty of the methodology, it is important to understanding how learners, educators and other stakeholders feel about SBL. Therefore, we conducted a formative qualitative assessment to describe the perceptions of key stakeholders towards establishment of simulation based learning and the potential role of simulation based learning in medical education at Mbarara University of Science and Technology, southwestern Uganda.

## Methods

### Setting

Mbarara University of Science and Technology (MUST) is a public university and is located 286 km, southwest of the capital city Kampala. Opened in 1989 the University’s Faculty of Medicine offers undergraduate training in Medicine, Nursing, Pharmacy, Physiotherapy and Medical Laboratory Science. The Bachelor of Nursing Science course (BNS) is a four-year course and Bachelor of Medicine and Surgery takes 5 years to complete. The University uses predominantly didactic approach through traditional lecturers, demonstration in the clinical skills laboratories, community placements at primary health care facilities and bed side teaching at the university’s teaching hospital.

### Study participants

The study participants included faculty members at MUST, specifically heads of department in the Faculty of Medicine, and members of the top administration at the University, and students enrolled in the Bachelor of Medicine and Surgery, and Bachelor of Nursing Science programs. We purposively selected the potential participants based on their key positions as stakeholders in teaching, learning and administrative decision making. We used key informant interviews for the administrators and department heads because these are a small group of people and were from different backgrounds of expertise and administration. In addition, each of them had considerable knowledge in the introduction and adoption of new teaching methodologies which would be best captured in an individual interview rather than a group discussion. We enrolled both medical and nursing students that had recently been introduced to SBL sessions at the newly opened simulation center, and those that had not been exposed at all to these sessions. And because the student numbers were larger, and due to our interest in examining group dynamics related to perceptions of simulation, we opted to use focus group discussions with them.

### Data collection methods

We designed an exploratory qualitative tool and administered this through Focus Group Discussions (FGD) and semi-structured key informant interviews (KII) to explore prior experience and perceptions towards SBL. We opted to use FGD due to large student numbers, and our interest in examining group dynamics related to perceptions of simulation. The tool included questions to identify the roles that faculty should play in the establishment of the simulation center and how or even whether this training should fit in the undergraduate medical education curriculum as well as determine the opportunities that the Simulation center may bring to MUST. We used this approach because medical simulation (MS) is a new teaching method at the University. Interviews and FGDs were conducted between the month of April and May 2017. Each KII lasted between 30 and 40 min. The FGDs comprised 7–9 students each and lasted for approximately 1 h.

Data collection was carried out by a trained and experienced researcher who was not a member of staff at Mbarara University and was not part of the simulation implementation team. Data were collected till the saturation point was reached (when no new information was generated in the FGDs and KIIs). We assessed the patterns, categories and variety of the new information generated to determine that saturation had been reached.

All interviews were conducted in English and were audio-recorded.

### Data analysis and coding

The audio files were transcribed verbatim into text and analyzed using thematic content analysis based on a priori themes generated from the research questions and emerging themes from the data. Data were analyzed manually. Three investigators (JN, CK and FB) read the transcripts and each independently generated codes from the transcripts and all authors reviewed the codes. We used two stages of coding; open and axial coding as has been described elsewhere [10]. At the open coding stage, we broadly explored perceptions from the stakeholders and examined features that were common in the data. At the second stage of (axial) coding, we used both inductive and deductive approaches to relate the together the codes that were generated in the first stage. The similar codes were grouped together to constitute or reflect the emerging patterns or themes i.e. thematic approach [11, 12].

### Human subjects issues

We submitted the proposal to Mbarara University Research Ethics committee and received approval. The study was registered at the Uganda National Council of Science and Technology. We obtained individual written informed consent from key informants and participants of the focus group discussions. We obtained written informed consent from all the study participants. Data were collected in private spaces with no intrusion. We ensured that all interview data were kept confidential by providing a unique identification number to identify the data. Only the participants’ identification number was included on the transcripts and audio files. All data were stored in password protected computer files and audio files were discarded after use. The interviews were carried out by an independent researcher who was not known to all participants. To ensure confidentiality of the respondents, particularly, given the small number of the key informants, we do not provide the specific job titles of the respondents.

## Results

We conducted 7 key informant interviews and these comprised representatives from the different clinical departments in the faculty of medicine namely Obstetrics and Gynecology, Pediatrics, Surgery, Internal medicine and the Institute of Maternal & Child Health. We also interviewed two representatives from the University administration.

We conducted three FGD with the undergraduate Medicine and Nursing students. One FGD comprised medicine students in year 4 and 5. The second one comprised year 3 nursing students and the third was a combination of both nursing and medicine students and the full composition is shown in Table 1.

**Table 1:** Composition of FGD participants

FGD #	Number of participants	Composition						
		Gender		Program		Year of study		
		Male	Female	Medicine	Nursing	III	IV	V
FGD one	9	5	4	8	0	5	0	4
FGD two	8	3	5	4	4	0	4	4
FGD three	9	2	7	0	9	0	9	0

### Emerging themes

Overall, findings were categorized into five broad themes: 1. Motivation to adopt simulation-based learning 2. Prior experience and understanding of simulation-based education 3. Outcomes arising from introduction of medical simulation; 4. Drawbacks to establishment of medical simulation; and 5. Potential remedies to the drawbacks; these are summarized in Table 2.

**Table 2:** Summary of results

Theme	Components
Motivation to adopt simulation learning	Limited opportunities to interact with patients
	Large number of students
	Potential breach of patient privacy
	Heavy clinical workload for teachers
	Modern method of learning
Prior experience and understanding	Significant knowledge of simulation
	Limited prior experience
	Early positive experiences among students
Outcomes arising from introduction of medical simulation	Visibility of the institution
	Income generating
	More skilled trainees
Drawback to establishment of SBL	Staff turnover
	Limited staff
	Sustainability of learning method
	Burdensome to the teachers
Remedies to the drawbacks	Curriculum integration
	Institutional funding for simulation

#### Motivation to adopt simulation-based learning

The study participants presented several challenges in current medical education and these ranged from the limited opportunity for students to interact with patients on ward and holistic patient management to limited exposure to rare cases in nature which on many occasions are left to the senior staff such SHOs (Senior Housing Officers). This often culminates into inadequate experiential learning.

*…‘touch’ [hands on] experience is limited, for medical trainees especially on the surgical ward. When we go to the field, we are expected to perform cesarean section and yet at year five, this is not done, we only assess patients. The PGs (Post Graduates) do the rest yet they are too many* ***(Medical Student FGD 2)****.*

The respondents noted there was a large number of students thus a large student to patient ratio which limits students’ full access to the patients. There were not enough opportunities to conduct clinical examination. The participants noted that having many learners practice repeatedly on the same patient negatively affects the privacy and well-being of the patients. The participants also mentioned students assessment was now inclined towards theory rather than practice to avoid repeat patient examinations. Students expressed concern of difficulty in finding a balance between theory and clinical practice.

*The resources are not there. By the fact that we are in a class of 78 and still cannot receive what we are supposed to be getting. The first years are 95 students so you can imagine such a number [when they get] in third year.. I don’t know how they will do it.* ***(Medical Student FGD 1)***

*Of course, one of the issues is that numbers have grown in the faculty. Being able to demonstrate these life experiences to students is hard because they are so many. In simulation everybody can be able to practice. So, it eases the teaching (****Male, Administrator****)*

The students also revealed that the clinicians were not always available for the students. They mentioned that many of them had to juggle between their clinical and teaching roles. The students usually rely on the Senior House Officers (SHOs) or postgraduate medical doctors for training yet these are equally occupied with their student roles. They also noted there was limited time allocated for student training on ward, specifically seven and a half weeks per rotation, which the students thought was insufficient. The students noted the requirement to have their log books signed overrode their interest to meticulously acquire the required clinical skills.

*…there is not enough time on the wards, we are given log books which are good but some of them are really big in that you seem not to focus on what you are going to learn but to fill it and get over with it. So instead of taking time doing a procedure and learning it, appreciating it; you are rushing it because you have to observe something else on another section to get a signature* ***(Medical Student FGD 2).***

Students articulated that such challenges often culminated into negative learning outcomes like limited confidence to handle patients, restricted exposure to rare cases, truncated team building and low student motivation. The students mentioned they were compelled to engage in poorly supervised clinical practice during term holidays, a practice they referred to as *‘quacking’* in order to acquire these required clinical skills.

#### Prior experience and understanding of simulation-based learning

Most students and key informants displayed reasonable knowledge of medical simulation as a method of teaching. Most of their descriptions fitted the broad definition; a new innovation in medical education involving the use of scenarios, models or mannequins to mimic “real life” situations rather than dealing with patients directly; which is the traditional mode of teaching. Although they did not have direct experience with SBL, they had picked up from external sources such as watching movies and television programs where these methods had been applied. Only one participant expressed lack of knowledge of specifically on what medical simulation is but alluded to the fact that it was an innovative teaching method for medical students.

*I would say medical simulation is trying to get people or lecturers embrace innovative ways of addressing the challenges that are related to maternal new born and child health* ***(Male University Administrator).***

Several key informants mentioned they had some prior experience with medical simulation. While some reported having participated in training at external institutions, they had on rare occasions practiced it while teaching students. Medical simulation is presently used in training in some skills in the nursing school, pediatric training under the Emergency Triage and Treatment (ETAT) program as well as the essential surgical skills course for the fifth-year students.

*For example, the surgical skills, it uses medical simulation. Instead of cutting real patients, students are taught to address certain procedures using animals, goats…., goats’ intestines and all that. It has been going on for some time* ***(Female Medical officer).***

The students and key informants who had recently been introduced to SBL also described their experiences at the newly established simulation center at MUST. Medical students from the nursing and medicine departments were introduced to practice simulation sessions. However, the time allocated for the exercise was felt to be short as the training lasted for only 2 days for students. Nevertheless, most students acknowledged that medical simulation provides a safe and comfortable learning environment for them. They mentioned the benefit of lack of fear of the potential negative patient outcomes which can occur when one is dealing with real patients directly. Simulation allowed them time to master a skill before directly handling a patient.

*I cannot panic that much. The first morning we came in, they read to us a scenario and trust me the adrenaline was very high but as time went on, they told us what should be done. We got used however much they were not the same scenarios as the first but as we did more; we got used and got to know what should be done say universally for emergencies since that’s what we dwelt on the most. There are some things supposed to be done to have the patient survive. There were basics that we got to know* ***(Student FGD One).***

The key informants also mentioned SBL has potential to provide an environment where there is limited concern about patient safety and the learners are confident. This was particularly important among the students initiating their clinical years. Simulation also allows for reflection on the scenarios, correct mistakes and hold discussions which may not be possible in a “real life” scenario in the wards where patients may need direct interventions. Working on patients’ hands on does not give time for reflection once confronted by the patient as mentioned in this excerpt;

*… I had given an example of a patient having acute asthmatic attack. You should have a way of having a model that simulates that asthmatic attack so as to see the signs and symptoms of asthma then the students study and practice what they are expected to do. After wards you sit down and reflect on what they have done well, what would have been done better and then may be repeat it to see if they have improved on performance* ***(Male Medical Officer)****.*

Further still, medical simulation allows sufficient time for students to get constant feedback especially during the scenario execution. Participants further reiterated that medical simulation was important for learning about or demonstrating rare conditions as well as conditions that are risky and emergency in nature. There are also certain conditions that cannot be practiced on patients; for example neonatal resuscitation. They mentioned that such practices were delicate and could only be taught through simulation. One student in the FGD described how their recent exposure to simulation enabled them recall and apply skills they had gained from the simulation session straight to a real patient on the ward in a case of post-partum hemorrhage.

*The important thing I learnt [from simulation] was identifying the danger signs. If I encounter such a case, I know where the alarms are going to arise from. I think it was the case [in the simulation scenario] of postpartum hemorrhage… I forgot that this person [mannequin] can bleed from a tear. There was something I did not do and the patient ended up in the operating theatre. The alarm signals are still ringing in my ears... As we were managing the case [of postpartum hemorrhage] on ward as an emergency, I remembered the alarm signals that came up in the management while in the simulation session. The mother had a cervical tear that was going way back into the uterus. The knowledge and teamwork I had learnt from simulation helped me and I enjoyed [managing the patient]. I am prepared if such a case comes up again* ***(Student FGD3).***

Teamwork was also reported to be a significant benefit of the simulation exercise and this encouraged active participation of all trainees in the simulation exercise. Students also reported that medical simulation promoted mutual respect between the instructors and the learners. Students were comfortable around their instructors, there was free exchange of information and they had the opportunity to spend more time together with their instructors unlike the “real life” clinic setting.

*When doing simulation in terms of teaching it was more effective than on ward. He had more time with us. He first corrected then told us what to do. It was better there. On wards at times we want to see how our senior does it, he is a team leader and is practicing what he preaches* ***(Student FGD one).***

*During simulation the lecturers were down to earth and were talking as if talking to a colleague and on ward or class you are a student and they shout and do whatever they want. Sometimes you feel intimidated and you cannot talk to them, they are like ‘gods.’ But in simulation they were so friendly, they would talk to you and you would be able to learn* ***(Student FGD Two).***

#### Outcomes arising from introduction of medical simulation

Overall, respondents were optimistic about the variety of opportunities and benefits that were likely to arise from the introduction of simulation based learning. There was anticipation among most of the stakeholders that being the first of its kind in the East African region, the simulation center would improve the visibility of the university. There was excitement this would attract more students to MUST. Respondents also expected the new teaching method would create more skilled doctors and nurses that will be widely recognized world over. There was also anticipation that the simulation center will bring in more partnerships which will support in building more capacity in medical simulation at the medical school

*If MUST medical school takes it up definitely the visibility of the medical school will go up and also by virtue of the skills acquired by the graduates since most medical schools in developed countries use it as a form of training. Our graduates would be widely accepted like their peers who are in other institutions where this is a practice. It’s like marketing the institution* ***(Male, Administrator).***

The simulation center was viewed as having great potential to generate income for the university through fees levied on external users of the center specifically in-service clinicians and medical students from other institutions within the region as well as attracting more grants into the university.

#### Drawbacks to establishment of medical simulation

There was concern about the possibility of staff turnover at the simulation center especially because these are high skilled staff and are difficult to replace. This was the same challenge presented for both the university staff and the project staff. There is limited expertise in the area and the few trained staff could leave for better positions in the region.

*We usually have a challenge with staffing both in number and at times the turnover. Some who are good may leave to go to other institutions or other environments so you lose people who are skilled and experienced and difficult to replace* ***(Male, University Administrator).***

There was concern about the sustainability of simulation center since it was starting with external funding, there is a need to ensure a steady flow of funding to sustain it. It was not clear whether the institution can commit to step in, in the event of lack of external funding. There was also concern about the maintenance of the equipment at the center as some of it has high end technology.

*SIM for life is coming in as a project and projects end and unless there is continual funding, its sustainability becomes a challenge so I think right from the start integrating it in the Faculty and University system for me it’s the best way of sustainability so that the external funding that comes in is just supplementing what is already in the university system and budget* ***(Male, Administrator).***

*It has been started with support from these collaborators. The only future would be that even if you have promises from these collaborators to support us now for the next 3-4 years, you cannot know whether support will be there after that period. Like any other project you have to be worried about the financial aspect for it to move to another level* ***(Male; Medical officer)****.*

Some respondents mentioned that some faculty may not be amenable to embracing a new form of teaching that they themselves did not undertake when they were students. They queried whether the level of acceptability of medical simulation would be high among all faculty, particularly those who were getting exposed to medical simulation for the first time. Further analysis of the stakeholders generated a new stakeholder group rather termed the ‘opposition’. These include groups of people or individuals anticipated to be important in contributing to the success of medical simulation but may not have buy-in into the use of medical simulation as a method of teaching. These were mainly teaching staff who are very loyal to the traditional methods of teaching and specifically those who do not believe that medical students can be taught using inanimate objects.

*…there are some members of staff who feel you don’t teach a doctor on a lifeless object and some who traditionally are opposed to the idea especially those that did not have this component in their medical training. They feel it’s not necessary and it gives the student a different impression of how patients should be handled. They feel some students may think you can handle a human being as a lifeless object* ***(Male, teaching staff).***

On the other hand, there was potential concern within some of the clinicians that medical simulation would engender a lack of seriousness among the students as they found it difficult to relay simulated scenarios with “real life” situations and thus not obliged to ensure patient safety.

*There might be a possibility that because I know I am working with a model, there is lack of seriousness […] they may think I can get away with anything […] During the scenarios and the debriefing the terms we use like being reminded that your patient’s blood pressure is reducing or the temperature is going up or the heart beat is up. It’s easy to work in such a scenario but you need to do something because if this was a real patient, how would I handle it? You can become relaxed and not take it too seriously; it can be tough. […] they tell you “oh a woman is bleeding… If I see real blood its better because it will make me run faster but now I am seeing a cloth that has been painted red, I may not take it serious* ***(Female Medical Officer).***

Some faculty members were concerned that the time table for teaching is already congested and that there would not be sufficient time allocation for medical simulation within the existing time table.

Some administrators were concerned that there might not be sufficient space for hosting the simulation laboratory. There was concern that there would not be enough space to accommodate the growing number of students in the Faculty of Medicine.

*As you know the university has a drive to increase the admission numbers. Already on the wards we feel the students we have are a bit many so if they increase, I don’t know what it could mean. I cannot be sure the space at the simulation center will accommodate these numbers* ***(Male, Teaching Staff).***

*The space we currently have is not enough to put us on that level. Even nationally I don’t think. It’s just small so we really need a better facility than what we have* ***(Male Administrator).***

### Potential remedies to drawbacks

Respondents suggested that attempts should be made to integrate medical simulation into the existing curriculum. They mentioned that this could be achieved if the Simulation center works with the individual departments to develop working guidelines. It was emphasized that the university could fund the simulation center to ease running of the activities including support for staffing and maintenance and not rely on external donor funding only. They suggested that the institution should put in place mechanisms that will ensure the sustainability of simulation-based learning even after external funding has ended.

### Conceptual framework

**Fig. 1:**
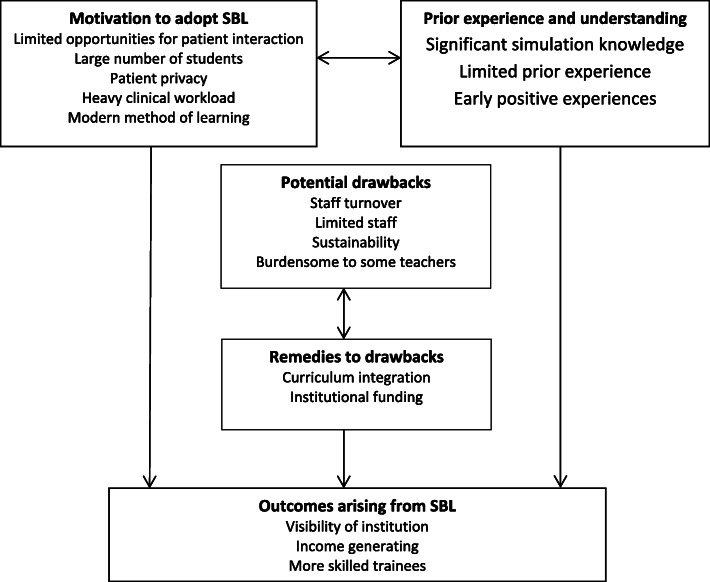
Conceptual framework to summarize the perception of stakeholders towards simulation based learning (SBL) and the potential role of the methodology in medical education at Mbarara University of Science and Technology, Uganda

The results are summarized in a diagrammatic conceptual framework represented below in Fig. 1. There is a set of motivating factors at the institution to adopt SBL and together with prior institutional experience and understanding of SBL, these factors may drive stakeholders to achieve the anticipated outcomes such as visibility of the institution and more skilled trainees. There are some drawbacks such as staffing and sustainability that need to be addressed in order to achieve successful implementation and the stakeholders suggested integration of SBL in the curriculum and institutional funding for SBL as remedies.

## Discussion

Our paper presents perceptions of stakeholders towards introduction of medical simulation at a medical school in a resource limited setting. Overall, there was optimism about the prospects of having a new method of teaching, which majority of stakeholders considered modern. They identified motivating factors which they anticipated might drive the uptake of SBL. The was significant knowledge about medical simulation but very limited experience among the stakeholders. The stakeholders identified many outcomes that might arise from the introduction of SBL but raised some concerns regarding its implementation.

In anticipation of the introduction of SBL, the stakeholders identified several factors that are likely to drive the uptake of SBL in medical education. On top of the list was the limited opportunity for medical students to interact with patients. This is primarily borne out of the growing number of medical students due to demand for more health workers in the region. Medical students now have to compete for patients to in order to learn how to take history, conduct clinical assessment and have their log books signed as evidence they have completed certain required tasks. Administrators and students all agreed that the current approaches to learning fall short of adequate. The findings in this study collectively underscore the key challenges faced during current medical education, unveil the motivation for new methodologies to deliver medical training and illustrate how medical simulation provides an important opportunity for alleviating these challenges to provide transformative learning as has been proposed elsewhere [8].

Although there was extensive knowledge of medical simulation among the stakeholders, there was very limited prior experience. Our simulation center is the first in the East African region, and therefore the limited experience was expected. There were many positive early experiences from the students who had attended their first simulation sessions at the center and they were eager to experience more. They felt that simulation provided a safe environment for them to practice and learn, and also engage in team work. These early experiences are reassuring and resonate well with the purpose of simulation-based learning. Our findings are in agreement with studies elsewhere among medical students where simulation was perceived well [13, 14].

The administrators and managers at the University saw some opportunities arising from the establishment of SBL in terms of increased visibility for the institution, a cadre of more skilled health workers and even income generating. Some of these opportunities such as visibility of the institution may be seen by simulation teachers as external to the primary purpose of SBL. However, such indirect benefits could be used as leverage by teachers to engage institutions to support the establishment of medical simulation. Simulation centers can also create courses that can be offered as an income generating service to sustain themselves.

Although majority of the study participants had favorable attitudes towards the use of simulation as a teaching and learning method, there were reports that some students would not take inanimate simulators seriously as they would with a real patient. There was also concern from some stakeholders that medical simulation involves mannikins and not real patients. This is an important finding and reminds teachers of SBL to emphasize that this method is not intended to replace traditional bedside teaching. It should be used as an avenue to prepare students for contact with real patients while providing a safe and controlled learning environment [15].

.Stakeholders were concerned about the sustainability of medical simulation, maintaining the simulation center and alignment SBL within the curriculum. Several participants suggested the need for integration of MS into the existing curriculum and the same has been suggested in previous assessments of SBL elsewhere [14, 15]. Even when start-up funding for medical simulation is external, integration works to complement the existing curriculum and eliminates the possibility of treating it as a standalone program. This also supports the sustainability and continuity of the teaching method at the institution. There was also concern regarding the staff turnover at the simulation center. This is an important concern and can be addressed by exposing as many faculty members as possible to simulation, so that dedicated simulation teachers based at the simulation center are relieved and roles of teaching are distributed. Our team developed a faculty development course to train as many faculty members as possible to address this potential challenge.

Our qualitative assessment has important strengths. We present novel data regarding perceptions toward simulation at an institution in a resource limited setting where this form of learning did not exist before. The lessons learned here are relevant not only for the Uganda, but for several institutions in sub Saharan Africa that plan to introduce SBL in the future. Our study provides insights into stakeholder perceptions that need to be considered before the introduction of medical simulation in a resource limited setting since most experiences in literature are of the developed world. Although the findings of the study are based on perspectives of stakeholders from one institution, we are optimistic that they can be transferred to other settings in sub Saharan Africa, where many institutions are grappling with increased demand for medical education and high skilled workers in the midst of shrinking resources. Our study has some limitations. We had less than ten key informants, but as top administrators and heads of departments, they already constituted a small group of individuals, hence the sampled number is representative of the leadership that we targeted. However, despite the small numbers, we were able to achieve saturation in data collection.

## Conclusions

In conclusion, our study has shown that there was significant buy-in from the institution, stakeholders are optimistic about the prospects of having a new method of teaching, which they perceived as modern, added to the traditional ones. There was significant knowledge but very limited experience of medical simulation. The stakeholders were optimistic to embrace medical simulation because of the potential to produce more skilled health workers and increase the visibility and reputation of the institution. There was some concern regarding the sustainability of simulation-based learning given that the funding to start was obtained from external donors. We recommend that other institutions with plans to initiate SBL conduct similar surveys or inquiries to acquaint themselves with their institutional landscape before implementation of SBL activities. The lessons learned in this evaluation are important for institutions to appreciate the unique features of their local environment as they seek to establish medical simulation as a learning tool for their medical students.

## Data Availability

The transcripts generated and/or analyzed during this study are not publicly available to protect the participants’ confidentiality but are available from the corresponding author on reasonable request and with approval from the Research Ethics committee at Mbarara University of Science and Technology.

## Abbreviations

SAFER: Stavanger Acute Medicine Foundation for Education and Research
SBL: Simulation Based Learning
CAMTECH: Consortium for Affordable Medical Technologies
FGD: Focus Group Discussion
MUST: Mbarara University of Science and Technology
ETAT: Emergency Triage and Treatment
MS: Medical Simulation
U of C: University of Calgary
SHO: Senior House Officer
KII: Key informant interview
